# ZYZ-772 Prevents Cardiomyocyte Injury by Suppressing Nox4-Derived ROS Production and Apoptosis

**DOI:** 10.3390/molecules22020331

**Published:** 2017-02-21

**Authors:** Ying Wang, Liangjie Zhong, Xinhua Liu, Yi Zhun Zhu

**Affiliations:** 1 Department of Pharmacology, School of Pharmacy, Fudan University, Shanghai 201203, China; tiantianwangying@126.com (Y.W.); 12211030038@fudan.edu.cn (L.Z.); liuxinhua@fudan.edu.cn (X.L.); 2 School of Pharmacy, Macao University of Science and Technology, Macao

**Keywords:** Apoptosis, Cardiac, Nox4, ROS

## Abstract

Nox-dependent signaling plays critical roles in the development of heart failure, cardiac hypertrophy, and myocardial infarction. NADPH oxidase 4 (Nox4) as a major source of oxidative stress in the heart offers a new therapeutic target in cardiovascular disease. In the present work, a novel flavonoid was isolated from *Zanthoxylum bungeanum.* Its structure was elucidated as Quercetin-3-*O*-(6′′-*O*-α-l-rhamnopyransoyl)-β-d-glucopyranoside-7-*O*-β-d-glucopyranoside (ZYZ-772) for the first time. ZYZ-772 exhibited significant cardio-protective property against CoCl_2_ induced H9c2 cardiomyocyte cells injury. In CoCl_2_ stimulated cardiomyocyte injury, ZYZ-772 inhibited expression of Nox4, and alleviated ROS overproduction. Importantly, ROS triggered MAPKs phosphorylation and P53 signaling mediated apoptosis were restored by ZYZ-772. Our findings present the first piece of evidence for the therapeutic properties of ZYZ-772 in preventing cardiomyocyte injury, which could be attributed to the suppression of Nox4/MAPKs/P53 axis. This will offer a novel therapeutic strategy for the treatment of cardiac ischemia disease.

## 1. Introduction

Apoptosis plays an important role in cardiac development and diseases [1,2], loss of cardiomyocytes by apoptosis even causes heart failure [3]. Many studies have demonstrated that reactive oxygen species (ROS) strongly induce cardiac myocytes apoptosis both in vivo [4] and in vitro [5]. High levels of ROS are generated from a variety of sources, including xanthine oxidase, NADPH oxidase, and the leakage of electrons from mitochondria [6]. Among them, NADPH oxidases are transmembrane enzymes dedicated to producing superoxide (O^2−^) by transferring an electron from NADPH to molecular oxygen and play important roles in cardiovascular pathophysiology. Nox4 as the major NADPH oxidase isoform expressed in the heart，is a principal source of oxidative stress [7]. Apoptosis was increased in transgenic mice with cardiac-specific overexpression of Nox4 compared to non-transgenic controls. Increased expression of Nox4 potently induces apoptosis in cultured cardiac myocytes, Nox4-induced apoptosis in cardiac myocytes was accompanied by cytochrome c release and prevented in the presence of Bcl-xL, suggesting that the mitochondrial apoptotic pathway is activated [8]. Now, Nox4 as a key molecule mediating oxidative stress and apoptosis of cardiac myocytes, it serves as an important target of heart failure treatment [9].

*Zanthoxylum bungeanum*, also called Sichuan pepper, is a common Chinese pepper growing widely in the area of Sichuan, Shanxi, Shandong, and Hebei Provinces of China. Apart from its common application as a condiment, *Z. bungeanum* is used for anti-microbial, improving digestion and analgesia in traditional chinese medicine treatment [10]. Until now, intensive investigation leads to isolate many classes of secondary metabolites from *Z. bungeanum* such as flavonoids, alkaloids, amides, lignans, coumarins [11,12]. However, the pharmacological effect of *Zanthoxylum bungeanum* was still poorly understood. In particular, cardio-protective effect of *Zanthoxylum bungeanum* and its key components have not been reported until now. Previous work in our lab found that *Zanthoxylum bungeanum* extract regulated the lipid metabolism and decreased lipid biosynthesis [13]. This motivated us to investigate what is the major bioactive component of *Zanthoxylum bungeanum* extract. Furthermore, the authors attempted to evaluate the cardio-protective activity of the isolated novel compound and reveal its molecular mechanisms.

## 2. Results

### 2.1. Quercetin-3-O-(6''-O-α-l-rhamnopyransoyl)-β-d-glucopyranoside-7-O-β-d-glucopyranoside (ZYZ-772) Protects against CoCl_2_ Induced Hypoxia Injury in H9c2 Rat Ventricular Cells

**Figure 1:**
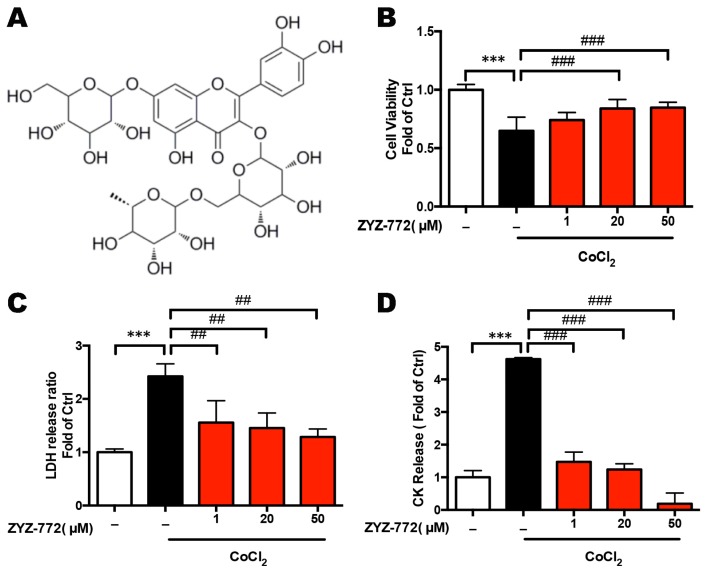
Analysis of the cardio-protective activities of ZYZ-772 in CoCl_2_-induced H9c2 cells. H9c2 cells were pretreated with/without different doses of ZYZ-772, followed by CoCl_2_ (1 mM) stimulation for 12 h. (**A**) Chemical structure of ZYZ-772; (**B**) effects of different doses of ZYZ-772 on cell viability in CoCl_2_-induced H9c2 cells; (**C**,**D**): effects of ZYZ-772 on LDH leakage and CK release in CoCl_2_-induced H9c2 cells. *** *p* < 0.001 versus control group. *## p* < 0.01; *### p* < 0.001 versus the vehicle group. Error bars represent the data obtained from the experiments repeated three times or more.

Quercetin-3-*O*-(6′′-*O*-α-l-rhamnopyransoyl)-β-d-glucopyranoside-7-*O*-β-d-glucopyranoside (Figure 1A) was isolated from *Zanthoxylum bungeanum* for the first time. The structure of this compound was established using ^1^H- and ^13^C-NMR (Nuclear Magnetic Resonance), DEPT (Distortionless Enhancement by Polarization Transfer), HMBC (1H detected Heteronuclear Multiple Bond Correlation), and MS (Mass Spectrometry) structural elucidation (Figures S1–S5). To explore the cardio-protective effect of ZYZ-772, Cobalt chloride (CoCl_2_) was applied to mimic hypoxic injury as reported [14]. As shown in Figure 1, CoCl_2_ induced hypoxic condition significantly decreased cell viability [(0.65 ± 0.11) vs. (1 ± 0.04)], meanwhile caused cell LDH [(2.42 ± 0.19) vs. (1 ± 0.05)] and CK [(4.62 ± 0.04) vs. (1 ± 0.17)] leakage compared with control group (*p* < 0.001). However, ZYZ-772 pretreatment restored CoCl_2_-induced decrease in cell viability [20 μM (0.85 ± 0.07), 50 μM (0.86 ± 0.43) vs. (0.65 ± 0.11)] and increase of LDH [1 μM (1.56 ± 0.33), 20 μM (1.45 ± 0.23), 50 μM (1.28 ± 0.12) vs. (2.42 ± 0.19)], CK leakage [1 μM (1.47 ± 0.24), 20 μM (1.24 ± 0.14), 50 μM (0.19 ± 0.27) vs. (4.62 ± 0.04)] in a concentration-dependent manner (*p* < 0.05; Figure 1B–D).

### 2.2. Activation of Nox4 Was Inhibited by ZYZ-772 in H9c2 Cells

**Figure 2:**
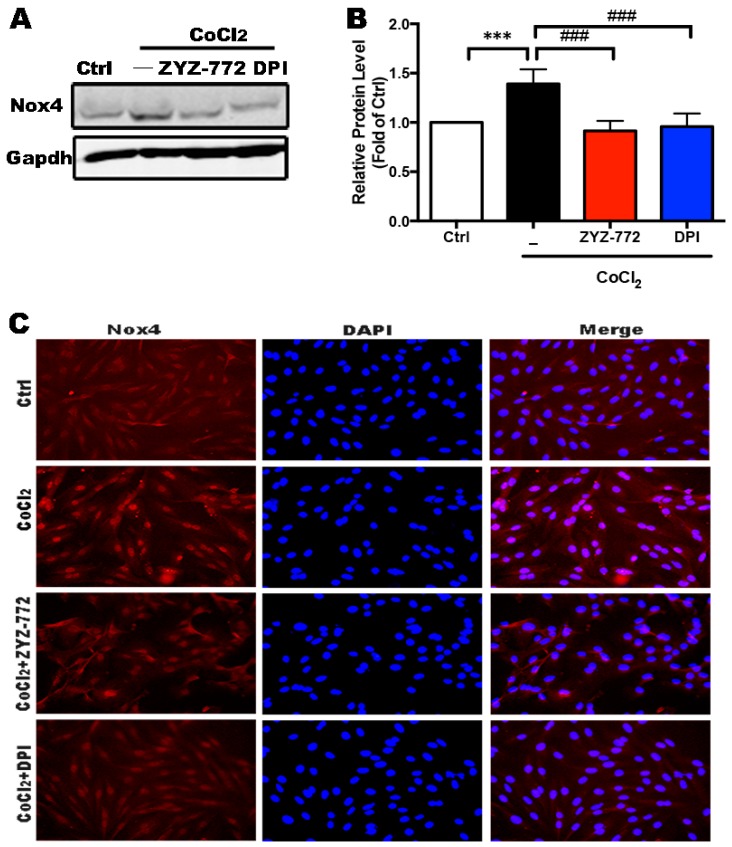
ZYZ-772 attenuated Nox4 expression induced by CoCl_2_. (**A**) Protein level of Nox4 was determined by Western blot. GAPDH was used as loading control; (**B**) statistical analysis of relative protein level of Nox4; and (**C**) Nox4 expression was displayed by immunofluorescence. Representative photomicrographs showing Nox4 expression (red) and DAPI staining (blue) indicates the nuclei. *** *p* < 0.001 versus control group. *^###^ p* < 0.001 versus vehicle group. Error bars represent the data obtained from the experiments repeated three times or more.

Nicotinamide adenine dinucleotide phosphate oxidase (Nox) proteins generate ROS in a highly regulated fashion and modulate several components of the heart failure phenotype [15]. Nox4 activation has been reported contributes to cardiac hypertrophy [16], myocardial infarction [17], and heart failure [7]. As indicated in Figure 2A,B, CoCl2 significantly increased NADPH oxidase 4 expression to 1.39-fold of control group (*p* < 0.001). However, this effect were restored by 50 μM ZYZ-772 [(0.91 ± 0.09) vs. 1] and Nox4 inhibitor diphenyleneiodonium (DPI) [(0.96 ± 0.12) vs. 1)]. A consistent result was observed with immunofluorescence of Nox4. Nox4 was located both at nucleus, membrane, and cytoskeleton. In Figure 2C, exposure to CoCl_2_ remarkably enhanced the fluorescence intensity of Nox4 (red), whereas, ZYZ-772 and DPI pretreatment alleviated this change of Nox4. These results suggest Nox4 was involved in CoCl_2_ induced cardiomyocytes injury and ZYZ-772 could effectively inhibit Nox4 activation.

### 2.3. CoCl_2_-Induced ROS Generation and MAPKs Signaling Phosphorylation Were Alleviated by ZYZ-772

**Figure 3:**
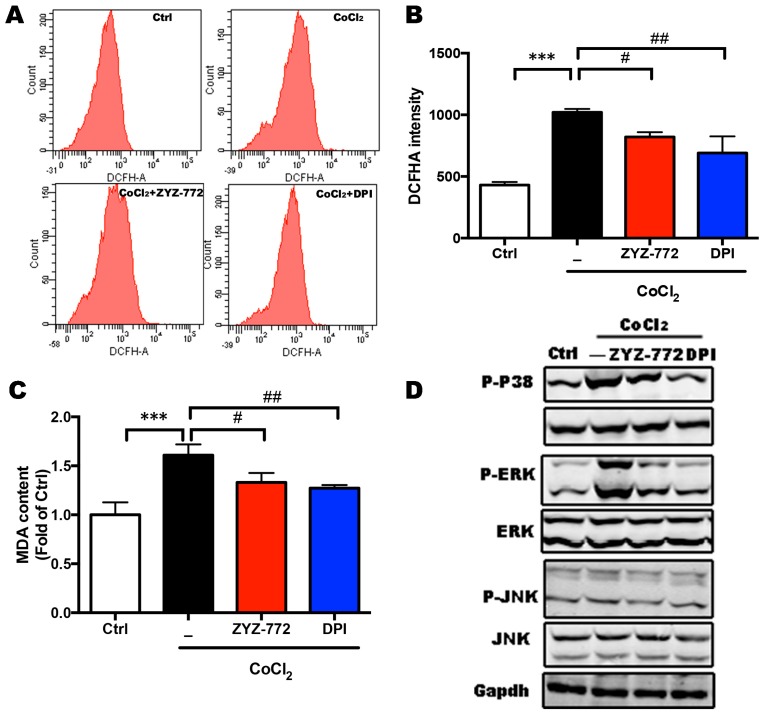
ZYZ-772 mitigated CoCl_2_ induced ROS generation and activation of MAPK signaling pathway. (**A**) Intracellular ROS detection by use of DCFHA probe, H9c2 cells were pretreated with/without ZYZ-772 and DPI, followed by CoCl2 (1 mM) stimulation for 4 h before incubated with DCFHA probe; (**B**) column bar graph of mean fluorescence intensity of DCFHA; (**C**) MDA contents of were measured with MDA kit; (**D**) phosphorylation of P-38, ERK, and JNK were determined by Western blot. GAPDH was used as loading control for cell proteins. Values are presented as mean ± S.D., *** *p* < 0.001 versus control group. *# p* < 0.05, *## p* < 0.01; versus vehicle group. Error bars represent the data obtained from the experiments repeated three times or more.

Nox4-mediated ROS generation was a key signaling pathway [18] involved in cell apoptosis which is responsible for a significant amount of the cardiomyocyte death that contributes to the development and progression of heart failure [19]. Consistently with the increase of Nox4, CoCl_2_ caused more than two-fold increase in intracellular ROS generation as monitored by DCFHA flow cytometry ((1020.33 ± 22.30) vs. (431 ± 19.61); (Figure 3A)), which was significantly decreased by ZYZ-772 ((821 ± 31.21) vs. (1020.33 ± 22.30)) and DPI ((690 ± 110.43) vs. (1020.33 ± 22.30)) (*p* < 0.05; Figure 3B). The production of MDA is an indicator of the development of lipid peroxidation and oxidative stress. MDA contents after CoCl2 exposure were also attenuated in ZYZ-772 group ((1.33 ± 0.08) vs. (1.61 ± 0.09)) and DPI group ((1.27 ± 0.03) vs. (1.61 ± 0.09)), (*p* < 0.05; Figure 3C). ROS and redox signaling triggers apoptosis by activation of upstream pro-apoptotic signaling pathways, such as activation of JNK and p38MAPK [20]. Obviously increased phosphorylation of ERK, JNK, and P-38 was observed after CoCl2 treatment (Figure 3D), whereas ZYZ-772 significantly antagonized these effects of CoCl_2_. As demonstrated above, novel molecule ZYZ-772 inhibited ROS generation and activation of MAPKs pro-apoptotic pathway.

### 2.4. Inhibitory Effects of ZYZ-772 on CoCl2-Induced H9c2 Cells Apoptosis

**Figure 4:**
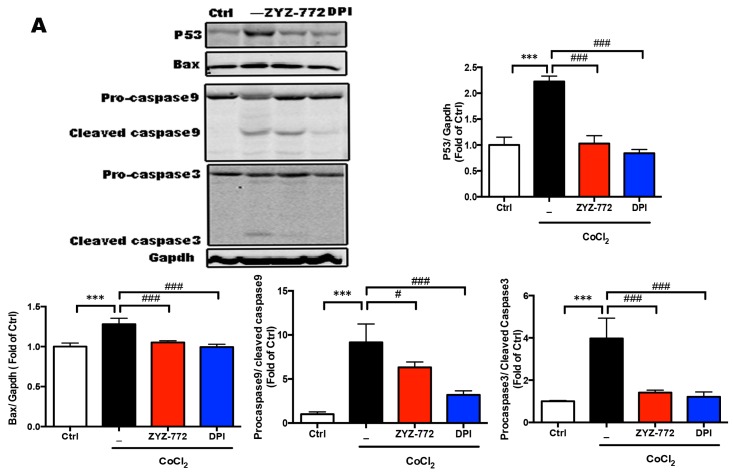

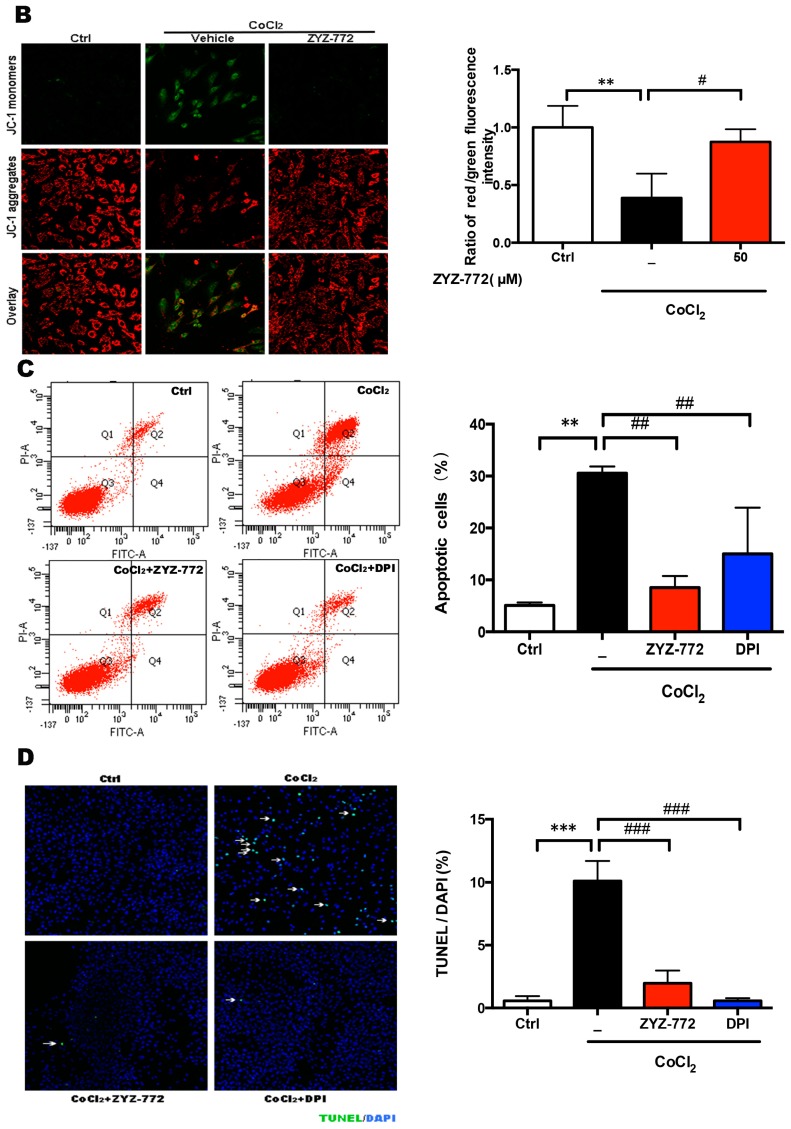
ZYZ-772 attenuates CoCl_2_ induced apoptosis via P53 mediated mitochondrial apoptosis signaling. H9c2 cells were pretreated with 50 μM ZYZ-772 or 10 μM DPI for 2 h, then stimulated with 1 mM CoCl_2_ for 12 h. (**A**) Gene expression of P53, Bax, Caspase-9 and Caspase-3 were measured by western blot analysis; (**B**) Representative image and quantitative analysis of JC-1 fluorescence staining for the changes in mitochondrial membrane potential (ΔΨm); (**C**) Flow cytometry detection of ROS with DCFHA fluorescence probe; (**D**) Immunofluorescent staining detection of apoptotic nuclei using TUNEL assay. Values are presented as mean ± S.D., ** *p* < 0.01; *** *p* < 0.001 versus control group. # *p* < 0.05; ## *p* < 0.01; ### *p* < 0.001 versus vehicle group. Error bars represent the data obtained from the experiments repeated three times or more.

It has been clear that activation of MAP kinase leads to the activation of P53-dependent mitochondrial apoptosis pathways [21]. Upon exposure to stressful stimuli, MAP kinases phosphorylate and activate p53, leading to p53-mediated cellular responses [22]. As exhibited in Figure 4A, CoCl_2_ induced the expression of pro-apoptotic protein: P53 ((2.22 ± 0.09) vs. (1 ± 0.12); *p* < 0.001), Bax ((2.22 ± 0.09) vs. (1 ± 0.12; *p* < 0.001) compared with Ctrl group. Meanwhile, obviously enhanced cleaved caspase-3 and cleaved caspase-9 expression was observed after CoCl2 treatment (*p* < 0.05). ZYZ-772 significantly antagonized these effects induced by CoCl_2_ by suppressed P53 and Bax expression. Then, the anti-apoptotic effects of ZYZ-772 were analyzed using the JC-1, Annexin V-FITC, and TUNEL staining. The loss of mitochondrial membrane potential (ΔΨm) is a hallmark for apoptosis [23]. Mitochondrial permeability transition has been implicated in the collapse of ΔΨm [24]. Changes in ΔΨm was monitored with the use of JC-1 probe. In Figure 4B, the control cells exhibited brightly stained mitochondria emitting red fluorescence (derived from JC-1 aggregates), whereas the CoCl_2_ stimulated cells mainly displayed green fluorescence (derived from monomers) indicative of mitochondrial depolarization and the collapse of ΔΨm. Therefore, the red/green ratio can help to identify a cell's mitochondrial membrane potential. CoCl_2_ stimulation caused approximately 62% loss of ΔΨm compared with the control cells. Conversely, ZYZ-772 pretreated cells showed a significant preservation of red/green fluorescence ratio in contrast with the CoCl_2_ group ((0.87 ± 0.09) vs. (0.39 ± 0.17); *p* < 0.05). Additionally, the apoptotic cell counts were measured using AnnexinV-FITC/PI by flow cytometry. Results in Figure 4C show ZYZ-772 drastically lowered CoCl2-induced proportion of apoptotic cells ((8.5% ± 1.8% vs. (30.6% ± 1.0%). Furthermore, DNA fragmentation, a characteristic finding of apoptotic cells [25], was visualized by TUNEL staining. Compared with Ctrl cells, CoCl2 increased TUNEL-positive nuclei (*p* < 0.001; (0.6% ± 0.3% vs. (10% ± 1.3%), but ZYZ-772 ((1.97% ± 0.82% vs. (10.1% ± 1.3%) and DPI ((0.57% ± 0.17% vs. (10.1% ± 1.3%) distinctly reduced TUNEL positive staining (*p* < 0.001; Figure 4D). As demonstrated above, ZYZ-772 strongly abrogated CoCl2 induced H9c2 cell apoptosis.

## 3. Discussion

Cardiovascular diseases are a leading cause of death worldwide, and it has been projected that cardiovascular deaths will increase from 16.7 million in 2002 to 23.3 million in 2030 due to aging populations [26]. Ischemic heart disease including acute myocardial infarction (MI) will remain an important cause of heart failure and mortality. The current standard of care for MI is early reperfusion of the occluded vessel with angioplasty or thrombolysis to reverse ischemia and increase the number of surviving myocytes. ACE inhibitors and beta-blockers are most used drugs to prevent remodeling after MI and progression to heart failure [27]. However, existing drugs are not efficient enough to reduce the disease burden as well as mortality. New drug discovery (NDD) in MI has become more challenging for last couple of decades [28]. In the last decade, a large number of pre-clinical studies have been published on the potential use of stem cells [29], microRNA [30], protein and peptide [26] drugs candidates for cardiac regeneration after MI. Despite the large number of novel therapies under basic scientific investigation, the translation of cardio-protective strategies to improve patient outcomes following myocardial infarction (MI) has been disappointing [31]. Therefore, there is an urgent demand for new drugs in this area to reduce the mortality and control the associated disability. Natural derived or originated compounds still play a major role as drugs, and as lead structures for the development of synthetic molecules. About 50% of the drugs introduced to the market during the last 20 years are derived directly or indirectly from small biogenic molecules [32]. The interfacing of biological and chemical assessment about natural products provides the best solution to increase the productivity in MI drug discovery and development. *Zanthoxylum bungeanum* (*Z. bungeanum*), named Huajiao in Chinese, is an aromatic plant of Rutaceae1. Isolation of many classes of secondary metabolites from *Z. bungeanum* such as flavonoids, alkaloids, amides, lignans, and coumarins have been reported before [11,12]. Zhang et al. isolated nine flavonoids from leaves of *Zanthoxylum bungeanum* and reported their antioxidant activity [10]. Qian Yang reported [33] anti-thrombotic effects of α-linolenic acid isolated from *Zanthoxylum bungeanum* seeds. Otherwise, the bioactivity evaluation of these compounds even *Zanthoxylum bungeanum* extract was fairly limited. Our present work reported a novel flavonoid isolated from *Z. bungeanum*: Quercetin-3-*O*-(6′′-*O*-α-l-rhamnopyransoyl)-β-d-glucopyranoside-7-*O*-β-d-glucopyranoside (ZYZ-772). Additionally, our salient results revealed for the first time that ZYZ-772 protected H9c2 cells from CoCl_2_ induced hypoxia injury and we further explored underlying mechanism of this cardio-protective function.

Reactive oxygen species (ROS) are proposed to contribute to the deterioration of cardiac function in patients with heart diseases [6]. Oxidative stress in heart failure or during ischemia/reperfusion occurs as a result of the excessive generation or accumulation of free radicals or their oxidation products. Unbalanced cellular redox status can impair signal transduction protein synthesis, enzyme activation [34], and lead ultimately to apoptosis. It has been suggested that Nox4 in cardiacmyocytes is a major source of mitochondrial oxidative stress, thereby mediating mitochondrial and cardiac dysfunction [7]. Given Nox4 a critical mediator in the pathologies of heart diseases, there are urgent needs to discover novel potential molecular diversity targeting Nox4. Here, CoCl_2_ treatment induced Nox4 gene expression and ZYZ-772 abolished the increasing of Nox4. Such effect of ZYZ-772 is similar to Nox4 inhibitor DPI indicating the potency of ZYZ-772 in regulating Nox4. Up-regulation of Nox4 enhances O^2−^ production and causes increase in oxidative stress. Correspondingly, our data suggested that ZYZ-772 reduced ROS generation and lipid peroxidation. A growing body of evidence has reported the mitogen-activated protein kinases (MAPKs) were activated by oxidative stress and that activation plays important role in apoptosis [6]. In our results, consistently, ROS elevated MAPKs phosphorylation in CoCl_2_ group and such effects were attenuated by ZYZ-772 and DPI. Upon exposure to CoCl_2_, MAP kinases phosphorylate and activate P53, leading to P53-mediated mitochondrial apoptosis that accompanied by up-regulation of pro-apoptotic proteins Bax and cleaved caspase (e.g., cleaved caspase-9 and cleaved caspase-3). However, ZYZ-772 was found to block P53-dependent apoptosis signaling. The anti-apoptotic capacity of ZYZ-772 was further confirmed as ZYZ-772 alleviated dissipation of ΔΨm and decreased apoptotic cells ratio.

In summary, we purified and characterized Quercetin-3-*O*-(6''-*O*-α-l-rhamnopyransoyl)-β-d-glucopyranoside-7-*O*-β-d-glucopyranoside from *Zanthoxylum bungeanum* for the first time. Pharmacological evaluation showed the compound possessed cardio-protective effects against CoCl_2_ induced hypoxia injury. Moreover, ZYZ-772 suppressed CoCl_2_ induced apoptosis via Nox4/MAPK/P53 axis. These results suggest ZYZ-772 with the great potential for treating cardiac disease targeting Nox4 mediated ROS generation and apoptosis.

## 4. Materials and Methods

### 4.1. Extraction and Isolation of Quercetin-3-O-(6''-O-α-l-rhamnopyransoyl)-β-d-glucopyranoside-7-O-β-d-glucopyranoside (ZYZ-772)

Using the heating reflux method, 15 kg of *Zanthoxylum bungeanum* fruit tissues were extracted × 3 in ethanol at 50 °C. The concentrated ethanolic extracts were suspended in H_2_O and successively partitioned with petroleum ether, ethyl acetate, and *n*-butanol. The *n*-butanol extract was found to have the highest hypocholesterolemic activity. Additional chromatographic separation was performed using a silica gel column (HSGF254, Yantai jiangyou silicone development Co., Ltd., Yantai, China) and using a chloroform-methanol gradient. Thirty-four fractions were collected and each assessed for biological activity with fraction 32 being determined to be the most active. Further chromatographic separation was performed on fraction 32 using macroporous resin (HP20, Mitsubishi Chemical Holdings, Tokyo, Japan) with a step gradient of composed of H_2_O/ethanol (from 9:1 to 0:1, *v*/*v*). The eluents were collected and pooled to produce twelve fractions (F32-1 to F32-12). Based on additional evidence for biological activity, F32-10 was subjected to column chromatography on a Sephadex LH-20 column and eluted with H_2_O/CH_3_OH (from 9:1 to 0:1, *v*/*v*) to give twenty three fractions (F32-10-1 to F32-10-23). F32-10-8 was purified using MCI gel (CHP20P/120P, Mitsubishi Chemical Holdings, Japan) and eluted with H_2_O/CH_3_OH (from 9:1 to 0:1, *v*/*v*) to afford the active compound (ZYZ-772). To increase the purity of ZYZ-772, ZYZ-772 was further depurated using a HPLC system [Agilent 1260 (Santa Clara, CA, USA), Waters SunFire Prep C18 (5 μm, 10 mm × 150 mm), (Waters Corporation, Milford, MA, USA)]. The structure of this compound was established using ^1^H- and ^13^C-NMR, HMBC and MS structural elucidation. ^1^H-NMR (400 MHz, MeOD) δ 1.085(d, *J* = 6.0 Hz, H-6′′′), 3.190–3.819 (m, sugar-H), 4.509 (d, *J =* 1.2 Hz, H-1′′′), 4.902 (s, H-1′′′), 5.291(d, *J* = 7.6 Hz, H-1′′), 6.217 (d, *J =* 2.0 Hz, H-6), 6.399 (d, *J =* 2.0 Hz, H-8), 6.910 (d, *J =* 8.4 Hz, H-5′), 7.562 (dd, *J =* 8.4, 2.0 Hz, H-6′), 7.678 (d, *J =* 2.0 Hz, H-2′). 13C-NMR (100 MHz, MeOD) δ16.333 (C-6′′′), 60.882 (C-6′′′′), 66.604 (C-6′′), 68.187 (C-5′′′), 69.601 (C-4′′′′), 69.752 (C-4′′), 70.590 (C-2′′′), 70.738 (C-3′′′), 72.392 (C-4′′′), 73.918 (C-2′′′′), 75.453 (C-2′′), 76.368 (C-3′′), 76.368 (C-3′′′′), 76.647 (C-5′′), 81.011 (C-5′′′′), 93.426 (C-8), 98.529 (C-6), 99.713 (C-1′′′′), 100.647 (C-1′′), 103.286 (C-1′′),104.094 (C-10), 114.672 (C-2′), 116.338 (C-5′), 121.505 (C-6′), 121.715 (C-1′), 133.367 (C-3), 144.392 (C-3′), 148.275(C-4′), 157.038 (C-2), 157.651 (C-9), 161.143 (C-5), 164.742(C-7), 178.079 (C-4). MS *m*/*z* 723.0 [M + H]^+^

### 4.2. Drugs and Reagents

Quercetin-3-*O*-(6′′-*O*-α-l-rhamnopyransoyl)-β-d-glucopyranoside-7-*O*-β-d-glucopyranosid (ZYZ-772) was isolated from *Zanthoxylum bungeanum*. Cobalt dichloride (CoCl_2_) was purchased from Sigma Aldrich (St. Louis, MO, USA). Inhibitor of Nox4 (diphenyleneiodonium) was obtained from Selleck Chemicals (Houston, TX, USA). The kits for, lactic dehydrogenase (LDH), creatine kinase (CK), lipid peroxidation MDA assay kit were obtained from Jiancheng Bioengineering Institute (Nanjing, China). The AnnexinV-FITC/PI kit, ROS kit, MDA kit, and JC-1 kit were obtained from Beyotime Bioengineering Institute (Lianyungang, China). Antibodies to P53 (2524), phosphor-Erk (4370s), Erk1/2 (4695s), phosphor-JNK (4668s), JNK (9258s), phosphor-p38 (4511s), and p38 (9212s) were from Cell Signaling Technology, Inc. (Danvers, MA, USA). Antibodies to GAPDH (MB001) were purchased from Bioworld (Nanjing, China). Antibodies to Nox4 (3187-1) was from Epitomics (Burlingame, CA, USA).

### 4.3. Cell Culture and Treatments

Rat embryonic ventricular myocardial H9c2 cells were obtained from American Type Culture Collection (Rockville, MD, USA). Cells were cultured in Dulbecco’s modified Eagle’s medium with 4.0 mM l-glutamine, 4.5 g/L glucose (Invitrogen, Carlsbad, CA, USA) supplemented with 1% sodium pyruvate, 1% penicillin/streptomycin, and 10% fatal bovine serum (Gibco, Grand Island, NY, USA) at 37 °C in a humidified atmosphere with 5% CO_2_. Cobalt chloride (CoCl_2_) was applied as a chemical hypoxia inducer. Cells were stimulated with 1 mM CoCl_2_ for 12 h to induce hypoxic condition in vitro. ZYZ-772 powder was firstly dissolved with DMEM to 2 mM, then diluted with DMEM to different concentrations (1, 20, 50 μM) before incubated with cells. For cell viability, LDH and CK assay cells were pretreated with different-dosed ZYZ-772 (1, 20, 50 μM) for 2 h prior to CoCl_2_ treatment. 50 μM ZYZ-772 and 10 μM DPI was used for the following molecular mechanism investigation experiments.

### 4.4. Cell Survival Assay

Cell viability was determined by the MTT assay. Briefly, H9c2 cells were incubated with ZYZ-772 at different concentrations for 2 h before possessing to the 1 mM CoCl_2_. After 12 h the medium was removed from the wells, added 100 uL MTT (0.5 mg/mL) to each well, and the samples were then incubated for 4 h at 37 °C. The formazan crystals were dissolved by adding DMSO, and the absorbance values were measure at 540 nm using microplate reader. Fold changes were finally used to indicate the data normalized.

### 4.5. LDH and CK Leakage Measurement

Both lactate dehydrogenase (LDH) and creatine kinase (CK) can be used as indicators of hypoxia induced cardiomyocyte injury. Therefore, the levels of LDH and CK in the supernatant were determined by CK and LDH detection kits (Nanjing Jiancheng Bioengineering Institute, Nanjing, China), according to the manufacturer’s instructions. The amount of LDH and CK were qualified by detecting the absorbance at 440 nm and 660 nm, respectively.

### 4.6. Western Blot Analysis

Cultured H9c2 cells were harvested by scraping and centrifugation, washed with PBS, and re-suspended in RIPA buffer. Soluble proteins were collected by centrifugation at 12,000× *g*. Protein lysates were subjected to 10% and 12% SDS-PAGE and transferred onto an NC membrane (Merck Millipore, Billerica, MA, USA). After blocking with 5% skim milk, the membranes were incubated with the respective primary antibodies in Tris-buffered saline (TBS) containing 0.1% Tween-20 overnight at 4 °C. The membranes were then incubated with the appropriate secondary horseradish peroxidase-conjugated IgG antibodies at a 1:5000 dilution (Proteintech Group Inc., Rosemont, IL, USA). Followed by incubation with the specific secondary antibodies. The reactive bands were detected by infrared fluorescence and exposed to the Odyssey Analysis System (LI-COR Biosciences, Lincoln, Nebraska, USA).

### 4.7. Assay of Mitochondrial Membrane Potential (ΔΨm)

ΔΨm was determined by an assay kit with JC-1, according to the manufacturer's instructions (Beyotime Institute of Biotechnology, Haimen, Jiangsu. China). JC-1 stains the mitochondria in cells with a high ΔΨm by forming red fluorescence J-aggregates, whereas in cells with depolarized mitochondria, JC-1 is present as green fluorescent monomers. In this way, mitochondrial depolarization was determined by a decreased ratio of red-to green fluorescence intensity by a fluorescence microscope (Zeiss Inc., Jena, Germany).

### 4.8. TUNEL Staining

DNA fragmentation was visualized by the TUNEL method using the TUNEL Apo-Green Detection Kit (Biotool, Houston, TX, USA). Cells were rinsed twice in PBS and fixed in 4% paraformaldehyde for 20 min. After being washed, cells were incubated with TdT terminal transferase and FITC-12-dUTP, and then counterstained with DAPI. After three washes, cells were observed under a confocal microscope (LSM 510, ZEISS, Jena, Germany). Apoptotic ratio was determined as number of TUNEL-positive cells to total number of nucleus stained. Six fields were included for each section and average number of three sections was used as percentage of apoptosis for each group.

### 4.9. Assessment of Apoptosis by Flow Cytometry

Apoptosis and necrosis were identified by means of double fluorescence staining with Annexin V-propidium iodide (PI). H9c2 cells (1 × 10^5^ cells per sample) were loaded with 5 μLPI and 10 μL Annexin V-FITC (BD Pharmingen, San Diego, CA, USA) at room temperature for 15 min in the dark. Flow cytometric analysis was performed using a flow cytometer (Becton, Dickinson and Company, Franklin Lakes, NJ, USA), after of Annexin-PI labeling.

### 4.10. ROS Assay

Intracellular ROS was measured using 2′7′-dichlorodihydrofluorescein diacetate (DCFH-DA) as a fluorescent probe (Beyotime Bioengineering Institute (Lianyungang, China). DCFH-DA is a non-fluorescent analog of fluorescein which will emit fluorescence after being oxidized by intracellular ROS. The bright fluorescence from the highly fluorescent DCF indicates the concentration and distribution of ROS. Cells were loaded with DCFH-DA (10 μΜ) for 30 min, followed by washing with PBS. DCF fluorescence was detected using a flow cytometer (Becton, Dickinson and Company, Franklin Lakes, NJ, USA).

### 4.11. Statistical Analysis

Results are expressed as mean ± standard deviation (SD). All data analysis was performed with the use of GraphPad Prism 6 software (GraphPad, Inc., La Jolla, CA, USA).

Differences between mean values of multiple groups were analyzed by one-way analysis of variance with Tukey’s test for post hoc comparisons. Statistical significance was considered at *p* < 0.05.

## Supplementary Materials

Supplementary materials are available online.
