# Supplementary material for: ZYZ-772 Prevents Cardiomyocyte Injury by Suppressing Nox4-Derived ROS Production and Apoptosis
Source: Molecules. 2017 Feb 21;22(2):331. doi: 10.3390/molecules22020331 (PMC6155929; doi:10.3390/molecules22020331)

Supporting Information

ZYZ-772 Prevents Cardiomyocyte Injury by Suppressing Nox4-Derived ROS Production and Apoptosis

Ying Wang[1,a], Liangjie Zhong[1,a], Xinhua Liu[a], Yi Zhun Zhu[ab*]

[a] Department of Pharmacology, School of Pharmacy, Fudan University, Shanghai 201203, China
[b]School of Pharmacy, Macao University of Science and Technology, Macao.
[1]These authors contributed equally to this work.
Corresponding authors Tel.: 86-021-51980208
E-mail: yzzhu@must.edu.mo or zhuyz@fudan.edu.cn

Table of Contents

Figure S1. MS analysis for compound **ZYZ-772** 2
Figure S2. ^{1}H-NMR (400MHz, MeOD) for compound **ZYZ-772** 3
Figure S3. ^{13}C-NMR (100MHz, MeOD) for compound **ZYZ-772** 4
Figure S4. DEPT (100MHz, MeOD) for compound **ZYZ-772** 5
Figure S5. HMBC (400MHz, MeOD) for compound **ZYZ-772** 6

Figure S1. MS analysis for compound ZYZ-772.

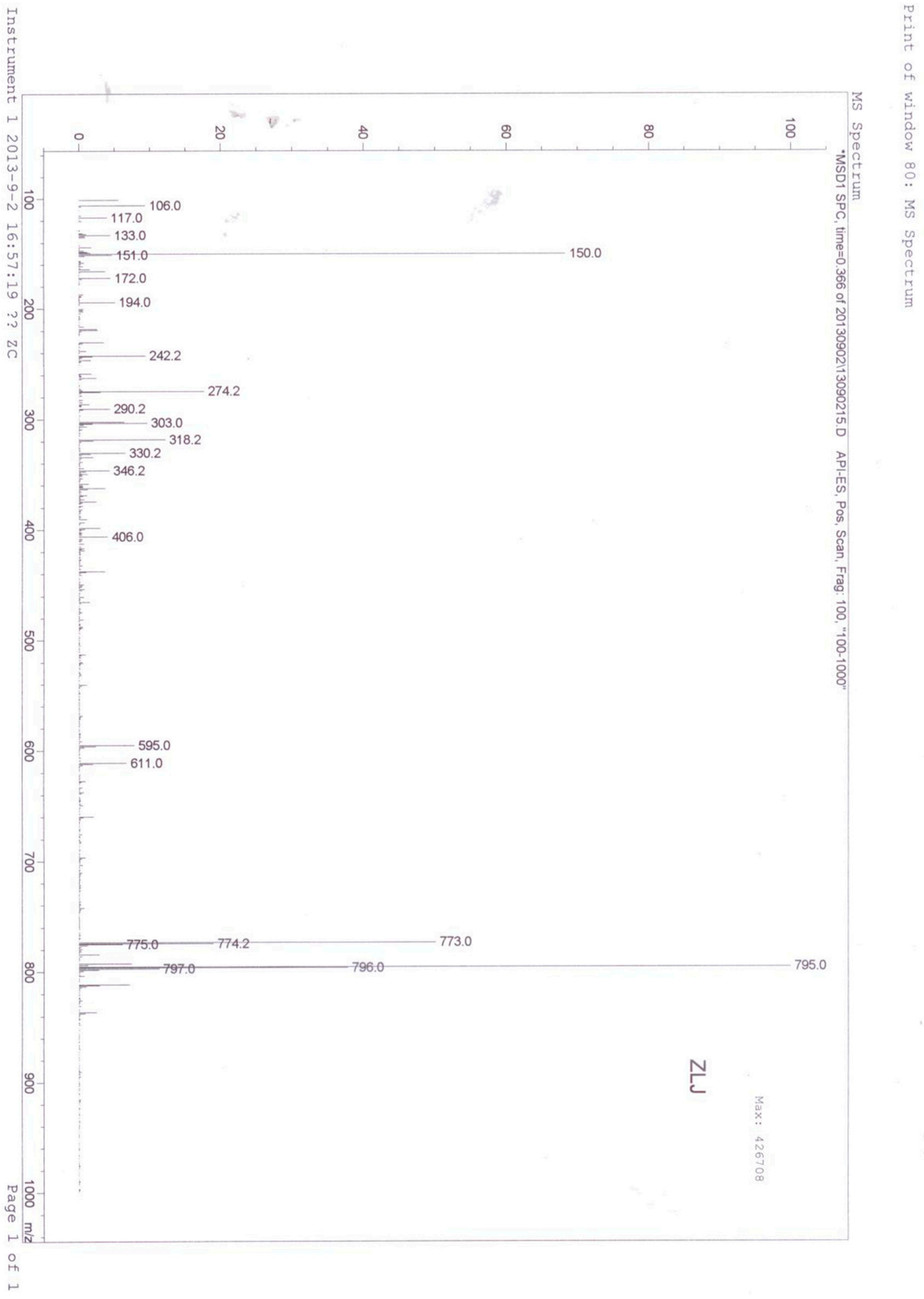

Figure S2. ^{1}H-NMR(400MHz, MeOD) for compound ZYZ-772.

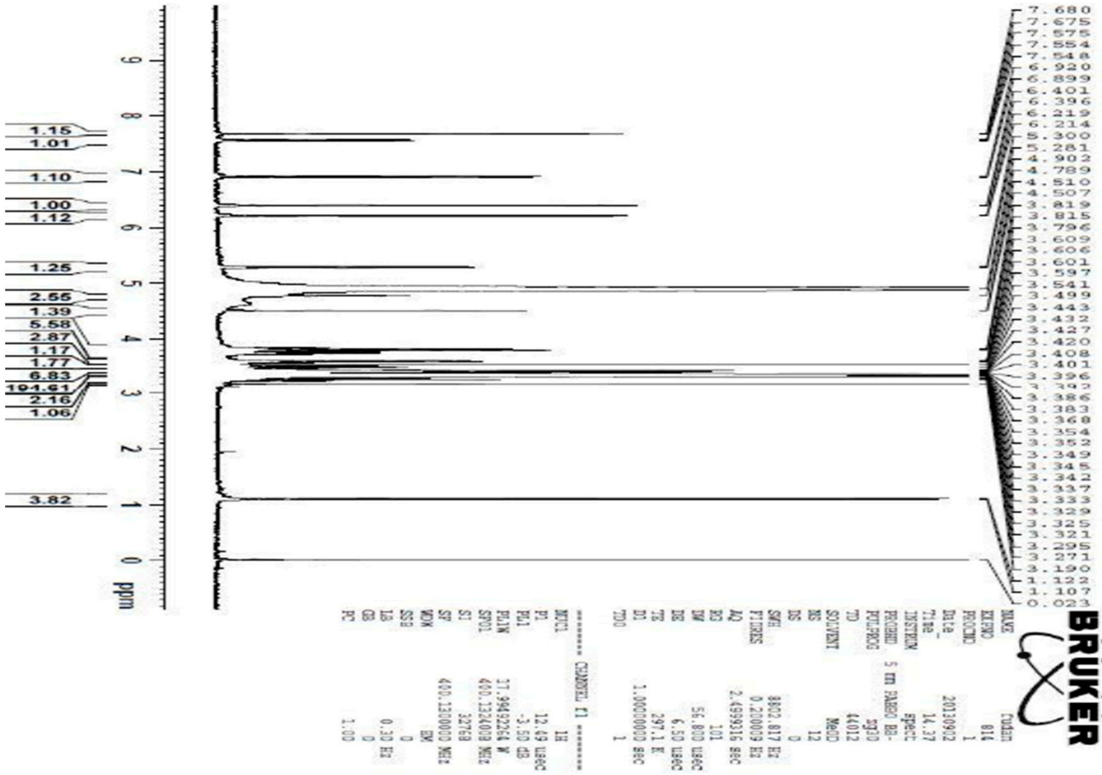

Figure S3. ^{13}C-NMR(100MHz, MeOD) for compound ZYZ-772.

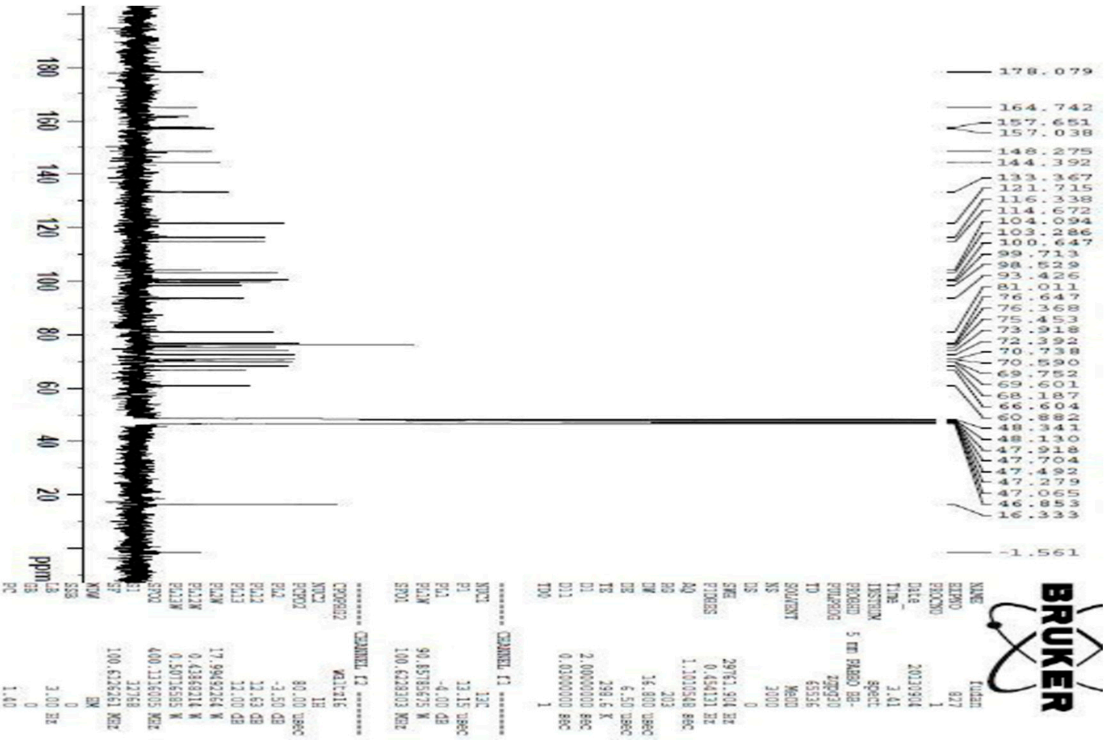

Figure S4. DEPT(100MHz, MeOD) for compound ZYZ-772.

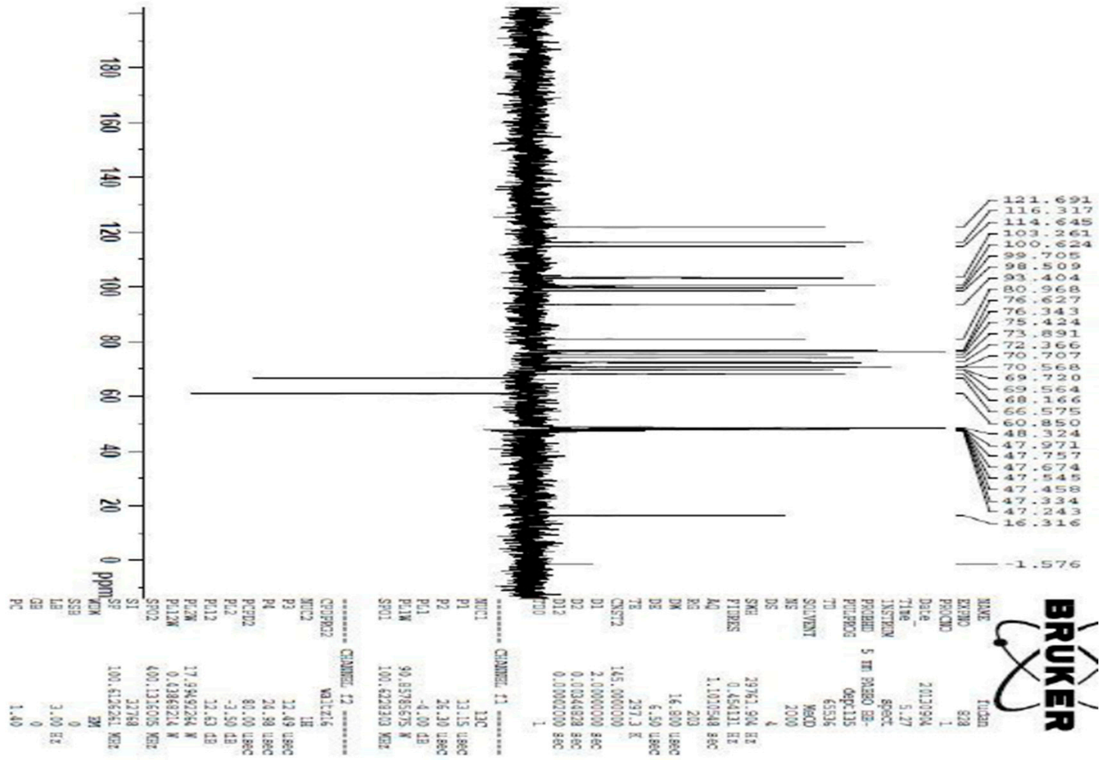

Figure S5. HMBC (400MHz, MeOD) for compound ZYZ-772.

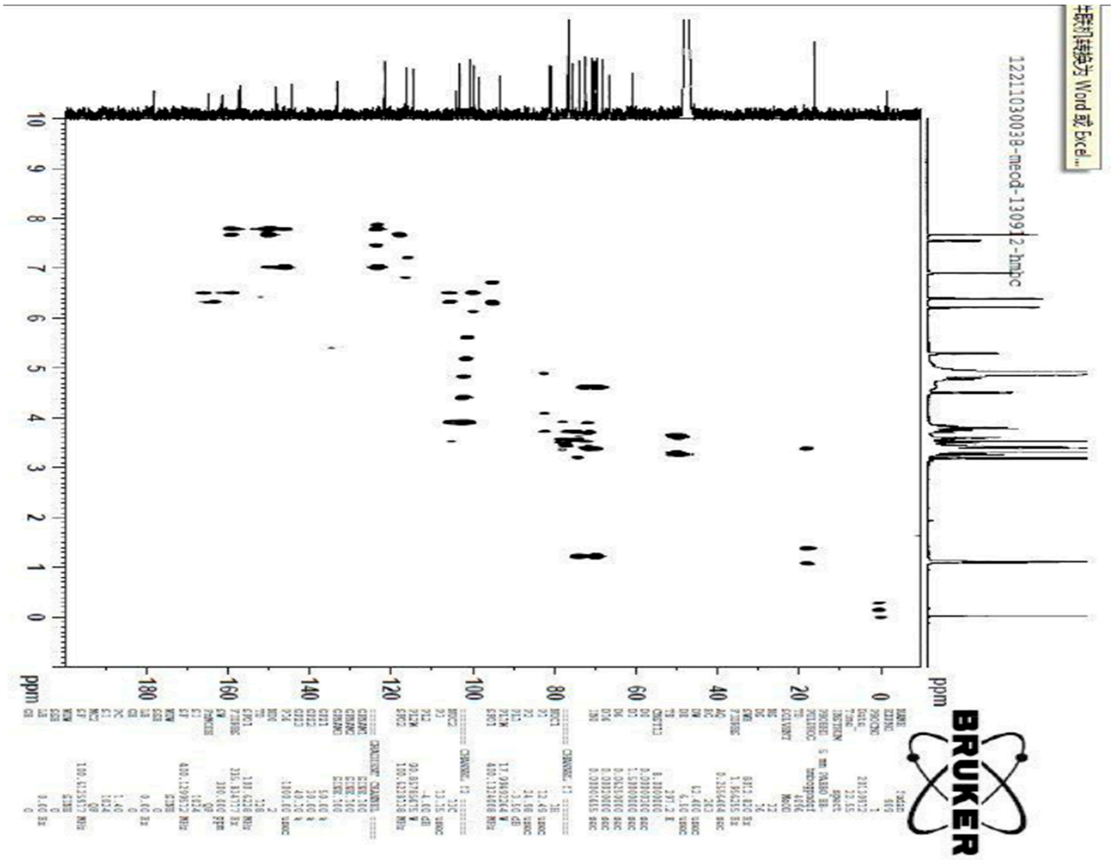

Supplement: Supplementary file 1 [file molecules-22-00331-s001.pdf]
